# Coping strategies for chronically ill children and adolescents facing the COVID-19 pandemic

**DOI:** 10.1590/0034-7167-2023-0045

**Published:** 2023-12-08

**Authors:** Lívia Lopes Custódio, Débora Cristina Couto Oliveira Costa, Cláudia Patrícia da Silva Ribeiro Menezes, Sarah Vieira Figueiredo, Julyana Almeida Maia, Maria Salete Bessa Jorge, Edna Maria Campelo Chaves, Ilvana Lima Verde Gomes

**Affiliations:** I Universidade Estadual do Ceará. Fortaleza, Ceará, Brazil; II Universidade Estadual do Piauí. Teresina, Piauí, Brazil

**Keywords:** Chronic Illness, Children, Adolescent, Psychological Adaptation, COVID-19, Enfermedad Crónica, Niño, Adolescente, Adaptación Psicológica, COVID-19, Doença Crônica, Criança, Adolescente, Adaptação Psicológica, COVID-19

## Abstract

**Objective:**

to understand the experiences and coping strategies of children and adolescents with chronic illnesses during the COVID-19 pandemic.

**Methods:**

a descriptive study, with a qualitative approach, carried out with six children and adolescents at the reception of an outpatient clinic of a pediatric hospital in the state of Ceará. Data collection took place from April to September 2021, using story-drawing, analyzed in light of Coutinho’s criteria.

**Results:**

two thematic categories emerged: Situations experienced by children and adolescents in times of COVID-19; Coping strategies for children and adolescents in their chronic illness process during the COVID-19 pandemic.

**Final considerations:**

understanding the experiences and coping strategies of children and adolescents with chronic illness demonstrated the expression of creative imagination, incorporated by subjective components, which brings to light an approximation with the reality perceived and interpreted in a context of the COVID-19 pandemic.

## RESUMO

**Objetivo:**

compreender as vivências e estratégias de enfrentamento das crianças e adolescentes com doenças crônicas durante a pandemia de COVID-19.

**Métodos:**

estudo descritivo, de abordagem qualitativa, realizado com seis crianças e adolescentes em acompanhamento ambulatorial de um hospital pediátrico do estado do Ceará. A coleta de dados foi realizada de abril a setembro de 2021, utilizando-se desenho-estória, analisada à luz dos critérios de Coutinho.

**Resultados:**

emergiram duas categorias temáticas: Situações vivenciadas por crianças e adolescentes em tempos de COVID-19; Estratégias de enfrentamento de crianças e adolescentes em seu processo de adoecimento crônico durante a pandemia de COVID-19.

**Considerações finais:**

a compreensão das vivências e estratégias de enfrentamento das crianças e adolescentes com adoecimento crônico demonstrou a expressão da imaginação criadora, incorporada por componentes subjetivos, que traz à tona aproximação com a realidade percebida e interpretada em um contexto de pandemia pela COVID-19.

## RESUMEN

**Objetivo:**

comprender las experiencias y estrategias de afrontamiento de niños y adolescentes con enfermedades crónicas durante la pandemia de COVID-19.

**Métodos:**

estudio descriptivo, de enfoque cualitativo, realizado con seis niños y adolescentes en la recepción de un ambulatorio de un hospital pediátrico del estado de Ceará. La recolección de datos se realizó de abril a septiembre de 2021, mediante dibujo-cuento, analizados a la luz del criterio de Coutinho.

**Resultados:**

surgieron dos categorías temáticas: Situaciones que viven niños y adolescentes en tiempos de COVID-19; Estrategias de afrontamiento de niños y adolescentes en su proceso de enfermedad crónica durante la pandemia de COVID-19.

**Consideraciones finales:**

comprender las experiencias y estrategias de afrontamiento de niños y adolescentes con enfermedades crónicas demostró la expresión de la imaginación creativa, incorporado por componentes subjetivos, que pone de manifiesto una aproximación con la realidad percibida e interpretada en un contexto de pandemia de COVID-19.

## INTRODUCTION

COVID-19, a disease caused by the new Coronavirus (SARS-CoV-2), caused several health challenges and led to the decree of a pandemic situation, as of March 11, 2020, by the World Health Organization^(1)^. Such a constant threat and widespread global advance has caused a high number of deaths.

The situation of the COVID-19 pandemic until the end of Epidemiological Week (EW) 50, on December 17, 2022, recorded 652,754,818 confirmed cases globally, with 6,664,842 deaths. In Brazil, during the same period, the country ranked second in number of deaths, totaling 691,863. In the local scenario, the state of Ceará had the highest mortality rate (305.3 deaths/100 thousand inhabitants) among the states of the Federation^(2)^.

Furthermore, in Brazil, 699 cases of deaths from Severe Acute Respiratory Syndrome were recorded in children and adolescents aged between six and 19 years. Among these cases, 329 were confirmed to be due to COVID-19 infection. Illness in this age group totaled 30,234 cases, of which 5910 were classified as COVID-19 in EW 50 of 2022^(2)^.

In view of this, health surveillance bodies urgently adopted guidelines and applied measures such as isolation of suspected cases and physical and social distancing in an attempt to minimize spread of disease and health service overload with positive cases^(3)^. At the peak of the first wave of the pandemic, seeking to contain the pandemic, which occurred in EW 30 (19-25/July 2020)^(4)^, measures were instituted with the stoppage of non-essential activities and the closure of some institutions (schools, stores, gyms and churches), avoiding the movement of people in public spaces.

Such measures had repercussions on people’s lives, changing their routines, locking them in and removing them from social life, due to the impossibility of face-to-face coexistence and physical contact limitation. This affected people’s daily lives around the world, making it necessary to find daily coping strategies to adapt to the conditions imposed at this time.

For this study, we chose the definition of Folkman and Moskowitz^(5)^, from the perspective of how people deal or what resources they use, or what their control capacity is to adapt to adverse circumstances. These strategies work as a dynamic process and, depending on the context, can be considered with different focuses. Coping can be classified into two focuses: on the problem (effort to try to change the source of stress, with the aim of modifying it) and on emotion (a response generated by the cognitive to reduce the emotional disorder in the face of a stressful situation).

Among those most affected, children and adolescents stopped attending school and coexistence with their peers and family was restricted due to mandatory confinement (lockdown), which led to health problems related to emotional and psychological issues, such as anxiety, depression, phobias, behavioral changes and increased sedentary lifestyle^(6)^. According to the Pan American Health Organization, during the pandemic, this population suffered severe impacts on both their physical and mental health, such as a worsening educational crisis and increased vulnerabilities, especially among girls^(7)^.

Faced with this, and given the new and specific context in which this public was inserted, coping strategies needed to be defined and carried out in different ways, becoming necessary depending on the situation and the stressor. When assessing the strategies adopted by Belgian children and adolescents (9-13 years old), social support through social networks, search for distraction, creation of a comforting atmosphere and search for information about the disease were identified^(8)^. Furthermore, one of the ways to observe children’s and adolescents’ emotions is through the technique of free drawings, such as Trinca’s story-drawing, used in this research^(9)^.

In the case of children and adolescents with chronic illnesses, identifying these strategies becomes even more valuable, since they already present changes, challenges and adversities in their daily lives due to the disease. Such an experience, regarding the health condition, may involve some risks or provide conditions of physical deficiencies or structural, developmental, behavioral or emotional disabilities, with long-term mental disorders, depending on the complexity, severity of disease and structures available to each person^(10)^.

Given the above, we sought to answer the following guiding question: how did children and adolescents with chronic illness experience the moment of isolation and social distancing and what coping strategies did they use in the face of the COVID-19 pandemic?

Thus, perceiving the child or adolescent as a chronically ill person includes the opportunity to provide the expression of their meanings or meanings attributed to the situation experienced in times of pandemic. Therefore, this study is necessary to understand the current reality, and it is believed that the data evidenced will contribute to favor reflection on how to promote adaptation strategies and adequate support for care that can favor promotional, preventive and assistance actions for psychological and physical well-being, in addition to mental health in coping with these or other pandemics.

## OBJECTIVE

To understand the experiences and coping strategies of children and adolescents with chronic illnesses during the COVID-19 pandemic.

## METHODS

### Ethical aspects

The study complied with Resolution 466 of December 12, 2012^(11)^, which deals with ethical issues involving human beings. All participants signed the Informed Consent and Assent Forms, and had their anonymity preserved, being identified with the letter C, for child, and A, for adolescent, followed by a numerical order (C1/C2/C3/C4, A1/A2).

### Study design

This is a descriptive study with a qualitative approach. In order to guarantee the validity of methodological aspects, this research followed the COnsolidated criteria for REporting Qualitative research (COREQ) checklist^(12)^.

### Methodological procedures

The story-drawing procedure, proposed by Trinca^(9)^, was used as a theoretical methodological framework as a possibility of welcoming and better understanding of children’s subjectivity, which can cover their lives, their history, their way of seeing and thinking about reality.

In this study, drawing was taken into consideration, as it is a symbolic representation that helps this child and youth audience in the performance and expression of feelings, thoughts and perceptions, in addition to being a facilitator for communication in view of data collection in research.

### Study setting

The study was carried out in an outpatient clinic specializing in the care of children and adolescents with chronic diseases, in a public pediatric hospital located in Fortaleza, Ceará, Brazil.

### Study participants

A total of eight children and adolescents were approached, of which six agreed to participate - four children and two adolescents (accompanied by a guardian) - and two refused (justified by indisposition). Children and adolescents aged 6 to 13 years (according to the age group monitored at the study site), of both sexes, diagnosed with at least one chronic disease, undergoing outpatient treatment/monitoring at the children’s hospital(at the time of data collection), who know how to verbally communicate their ideas and present psychomotor development to carry out the drawing, were included. Those who had difficulty understanding the instructions and/or expressing their ideas in order to develop a consistent representation style between graphic production and spoken word were excluded.

### Data collection and organization

The study took place from April to August 2021, using a pilot test with three participants, whose data were discarded. The sample was used for convenience, not probabilistic. Participants were approached in person, after acceptance and approval from their legal guardian as well as children or adolescents to participate in the research. The selection for data collection occurred until the information regarding the objectives outlined was reached.

The information was collected by a researcher, psychologist, doctoral student in public health and who, trained in qualitative studies, revealed her interest in investigating the emotions experienced in adverse times of the pandemic. Initially, direct contact with children and/or adolescents’ guardians occurred in the waiting room before consultation, with a brief presentation of their resume and the objective of this study.

Then, participants were directed to a specific room for interviews in the outpatient clinic, which took place at a single moment. A self-constructed form was applied to the adult responsible for the participant to characterize the sample regarding age, origin, type of chronic illness and education. The second instrument used was Trinca’s story-drawing^(9)^. For the story-drawing activity, materials were provided to children and adolescents, such as black pencils, A4 sheets of paper, clipboard, box of colored pencils with twelve units and colored pens.

Participants were asked to create a drawing based on the following instruction: “Draw about what it is like for you to live with a chronic illness in times of the new coronavirus pandemic”. After the production was completed, they were invited to talk about what they had drawn, either in the form of a story or an explanation, in order to attribute its meaning or meaning verbally. At that moment, the researcher recorded, in written form, the information verbalized by participants. This application took place in a single moment, without the need to repeat interviews, freely, individually, with an average duration of 30 minutes.

Data (written and/or verbalized productions) were transcribed into Microsoft Word, observing speeches obtained during the records in field diary, without recordings (audio or visual). The drawing was used as a communication tool, focusing on verifying symbolic elements that were significant during data collection.

### Data analysis

Content analysis was used for drawings based on the criteria described by Coutinho^(13)^, in its five stages, namely: systematic observation of drawings and themes; text skimming of story contents; selection of drawings based on graphic similarities and/or approximation of themes; material exploration; and identification of meaning cores, categorization and treatment of results.

The written and verbalized productions were subjected to content analysis using the webQDA software^(14)^. The data, which were previously transcribed into Word, were inserted into the system from internal sources through automatic import. In the focus process, data related to children and adolescents with chronic illnesses and their experiences during the COVID-19 pandemic were automatically coded (home, no play, problem focus, spirituality focus, emotion focus) using descriptors, which enabled the construction of empirical categories through coding tree.

Drawing was taken into consideration as a communication tool, with a focus on verifying symbolic elements that were significant as well as the possible complementation of post-drawing narratives. Forms, content and harmony were considered as elements that could contemplate the analysis of content conveyed.

## RESULTS

### Participant characterization

The sample comprised four children and two adolescents aged between six and 13 years old. Among the participants, five were female and one male, living in the Ceará cities of Itapipoca, Granja, Pacatuba, Quixeramobim and Fortaleza. Regarding chronic diseases, cancer (C1), myelomeningocele (C2), heart disease, urological disease and tumor (C3), pneumonia and asthma (C4), sickle cell anemia (A1) and kidney disease (A2) were identified.

During the application of Trinca’s Story-Drawing^(9)^ instrument, it was noticed in the feedback (testimonials) that the proposal resulted in positive movements by participants in the development and expression of their activity.

In this way, the themes were derived from the productions inherent to data collection, which emerged in two thematic categories: Situations experienced by children and adolescents in times of COVID-19; and Coping strategies for children and adolescents in their chronic illness process during the COVID-19 pandemic.

### Situations experienced by children and adolescents in times of COVID-19

Reading the drawings allowed the researcher a psychological understanding of participants’ reality, who outlined their anxieties, fears and perceptions of reality during the pandemic, listed in the first category. Chart 1 presents a report on the feelings of some participants and drawings in relation to situations experienced by participants during the COVID-19 pandemic.

**Chart 1:** Situations experienced by participants during the COVID-19 pandemic. Fortaleza, Ceará, Brazil, 2022

Drawing description	Participant’s speech	Drawing
Participant (C1)* drew himself inside the house, where the door would be. He placed an X, and, next to the house, a bicycle.	*This is me, I’m super sad, oh, because I’m at home, unable to go out, unable to do what I love most in my life, which is playing. I live stuck at home. I can’t play or ride a bike* [...] *stay at home. This is very annoying. No, I couldn’t even walk. I was dying to go after my friends, but my mother wouldn’t let me. Before, I rode my bike every day, played with my friends, now I can’t. I hope this* (disease) *goes away soon. Worst thing in my life.*	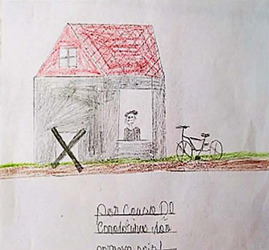
Participant (C2)* made a more colorful drawing, looking like two representations. He marked an X on the windows, as something forbidden. The participant initially pointed to the largest house, at the top of the sheet, and made the first report. Then, with a slightly saddened look and voice, he pointed to the bottom of the sheet, showing the drawing around the smaller (blue) house.	*That’s where I live. My house. But now I’m “inside” the house and I can’t play. Do you see it’s closed? Well, my mother closes it so no one can go out* [...] *I spend a lot of time alone at home. Playing is good or nothing. I only live there* (house). *Here, before it was me, it was like this, I only played with my friends. Now you can’t. I liked it a lot* [...]. [...] *I made these lines in black in this part, because I can’t play with them* (other children) *anymore, so I have to stay at home.*	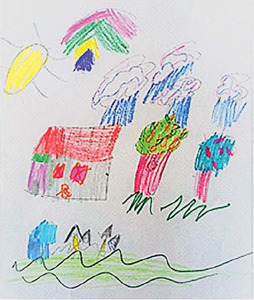
Participant (A1)* drew himself inside his house, holding a piece of paper in one hand and a pencil in the other hand. Doors and windows are closed, and, next to the house, a tree appears, and on the other side there is a lamppost.	*Inside this house, it’s me, I’m inside it, because I couldn’t leave. Not just me, but everyone* [...] *I even had to study at home. I couldn’t even play. I wrote in this sentence below: “During the pandemic, I stayed at home more than I went out”.*	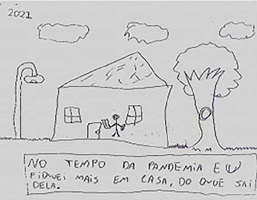

*C - child; A - adolescent.*

### Coping strategies for children and adolescents in their chronic illness process during the COVID-19 pandemic

Regarding coping strategies, it was noticed that they were different for each participant. While one expressed that turned to their spirituality, with the exposure of an angel (focus on spirituality, emotion), another brought the use of their skills to face situations during COVID-19 (focus on the problem), and another presented their family (focus on emotion) as a form of recurrence, as shown in their drawings. On the other hand, other participants (C1, C2 and A1) did not demonstrate coping strategies, either in their speeches or in their drawings.

Chart 2 presents the materialization of the feelings of some participants through drawings in relation to coping strategies in crisis situations in children and adolescents in their chronic illness process during the COVID-19 pandemic.

**Chart 2:** Coping strategies in crisis situations for children and adolescents in their chronic illness process during the COVID-19 pandemic. Fortaleza, Ceará, Brazil, 2022

Drawing description	Participant’s speech	Drawing
Participant (C3)* sought a way to portray his feelings in his spirituality. He drew an angel above his head.	*Here it is me and the angel of Jesus, above me. It is killing the coronavirus. He’s giving me protection, so I don’t catch the virus. I ask Jesus to protect me. I always do this and it works.*	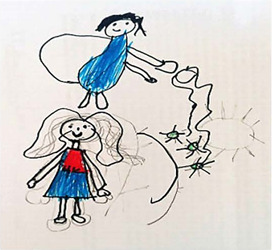
Participant (C4)* drew his family holding hands inside a heart (protected). The virus was represented outside the heart.	*I drew my whole family. There’s my aunt, godfather and cousins here, but there’s also my father, my mother and my brothers. We are covered in the heart that protects. The family is all wearing masks to protect themselves. Heart to protect, it is made of stone, it is hard. Nobody can open it. It’s tough as a rapadura* [...] *COVID, in green, is out of the heart* [...] *this is the sun* (yellow), *it is sad because it wanted to walk and destroy the coronavirus, but it couldn’t.*	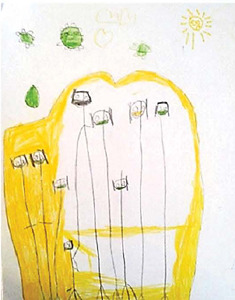
Participant (A2)* chose to portray two moments: the drawing above depicts a moment of domestic activity in the kitchen of his home and below the drawing next to the tree in the backyard.	*During the pandemic, I stayed at home more, so I wanted to read and learn how to cook. There was nothing to do* [...] *I learned how to make couscous in a frying pan, omelet, oatmeal cookies and other foods. I too got sick and couldn’t eat anything. I searched on the internet and did it. For me, it was cool. Another good thing was that I learned to read, because I didn’t like it, but it wasn’t a school book, no, it was a book with other readings. I knew I didn’t like it before. Even my mother got excited* (laughs); *she bought two books for me and I read them. That’s what I did most during the pandemic.*	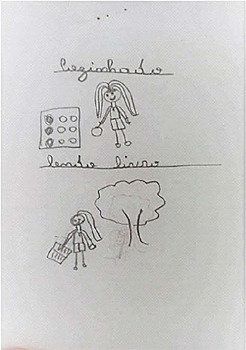

*C - child; A - adolescent.*

## DISCUSSION

The requirement to stay indoors as part of social isolation recommendations was imposed by health surveillance bodies to prevent, control and minimize the spread of COVID-19 at local and global levels^(3,15)^. This became one of the most efficient non-pharmacological interventions, since at the time, a period of high mortality (early 2020), there were still no specific vaccines for the disease^(16)^.

In this scenario, the present study observed that the experiences and coping strategies, in crisis situations of children and adolescents with chronic illnesses during the COVID-19 pandemic, were expressed through oral reports and drawings. It was possible to notice similarities in relation to participants’ experiences during the COVID-19 pandemic, as seen in the first category, described in Chart 1.

Participants’ representations were lived positions similar to the reality found by a large part of the world’s population, when there was a need to be indoors during the peak period of the first wave of the pandemic. These positions were observed, mainly, in the drawings of C1 and C2, in which an X-shaped representation was expressed on the door and windows, resembling a “no exit or entry” sign. Staying at home generated a feeling of enclosure, prison or confinement between these two participants.

The negative emotion associated with widespread confinement through quarantine was demonstrated with the same similarity in another study, carried out in Wuhan, which pointed to loss of control and the feeling of being trapped due to several factors, such as an increasing number of new cases, severity of the situation and imposition of measures^(17)^.

In addition to the issue of having to stay at home, combined with the need to comply with social isolation measures, this research also reached another important aspect in participants’ lives: the perspective of no longer being able to play, remembered from drawing samples and complementation of speech excerpts, which were quite expressive by A1, C1 and C2.

Speeches show impediment to doing activities outdoors, not indoors, demonstrating the desire to have a more active and apparently more dynamic lifestyle, with the need to explore what is outside, touch objects, nature and interact with other children. The opposite was noticed by Souza *et al*.^(16)^, in which children reported that, even in times of quarantine measures, they continued playing, whether at home and/or in the family context.

Still as described in the literature, in some games, participants included the virus and the changes they went through in their lives during this period, for example: experiences of games such as cleaning doll hands to prevent them from being contaminated; playing doctor to diagnose the siblings, whether or not it was COVID-19; In other types of games, the virus always acted as an evil villain^(17,18)^.

Social isolation possibly did not prevent children from playing, but it challenged them to modify the habits used in playing, including imaginary and “pretend”. Such experiences or reactions demonstrate that children reenacted the traumatic event by including the coronavirus character in their games, relating it to what happened or how it was absorbed into their life experiences.

Still from the perspective of playing, it appears that children construct their social and historical meanings, explore the environment, establish social relationships and stimulate their imagination and intellect. Through play, there is a process of building knowledge that can help balance the tensions arising from their cultural world for the subsequent construction of their individuality, personal brand and personality^(19)^.

As it is a comprehensive and new theme, this research made it possible to introduce another perspective, coming from participants’ personal experiences, which includes coping strategies in the face of crisis situations in their chronic illness process during the COVID-19 pandemic, described in the second category.

Such coping strategies are similar to those evidenced in a study carried out in the United Kingdom with young people and adults during the COVID-19 pandemic, in which participants revealed ways of coping with a focus on socialization and support among family members, being facilitated not by experiences of physical approach, but through video calls. Another strategy included participation in meditation activities, with a greater focus on spirituality, and also included exercise and healthy eating^(18)^.

On the other hand, research that analyzed the emotional repercussions of the pandemic for adolescents in São Paulo (Brazil) observed that the main adaptive resource used by participants was distraction with activities via the internet and social networks, based on adolescents’ own initiatives^(20)^. These aspects differed from the present research, in which the use of the internet was not mentioned by adolescents and children, which may be related to participants’ socioeconomic level and access to digital technologies.

In this sense, another study in the Republic of Korea and the United States also highlighted the importance of using online activities as a problem-focused coping strategy, through searches for creative and cultural actions during the pandemic in museums, galleries and other educational entities virtually. It is worth noting that it was parents who sought out these virtual event arts and crafts activities for learning, making an investment in time spent with their children, occupying them and providing some educational value^(21)^.

Therefore, it is important to highlight the valuable role of family members, health professionals as well as the support network for children and adolescents, in order to help them in this process of facing the difficulties highlighted in a global crisis situation through guidance, support and encouragement to actions that contribute to alleviating their suffering, especially in the context of social isolation. Relying on family support was also one of the forms of coping evidenced in the present research, through A2’s speech, in which the mother, upon realizing the awakening of reading, provided books to encourage her daughter.

The absence of such guidelines and attitudes that welcome these individuals, providing them with conditions to implement adaptive measures that make them feel better, can act as an obstacle in the elaboration and development of effective strategies to improve their well-being, contributing to the maintenance of their suffering^(20)^.

In agreement with other studies^(22,23,24)^, it was observed that children and adolescents used strategies focused on spirituality, an expressive way to encounter the feeling of love, comfort, support and protection from God, cooperating to cope and adaptation to stressful situations, including when it comes to health, care and humanization. The focus of spirituality was evidenced in a positive and adaptive way in this study, functioning as an active and significant resource associated with psychological well-being in various difficult circumstances and even in different countries, through the ability to deal with stressful situations.

However, it should be noted that, in general, there is no order or classification framework in relation to coping strategies, in a simplistic attempt to affirm them as good or bad, adaptive or maladaptive, as they can be changed according to over time and stress^(25)^. The way to face a stressful situation, at any stage of a person’s life, involves subjective issues, availability of resources and effort, in an attempt to manage or deal with a stressor.

Furthermore, the effects of the pandemic, together with the situation of isolation, drastic changes in people’s routines and the threat of contracting the disease, can have a psychological impact^(26)^. Emotional reactions and behavioral changes have been solidly evidenced in the literature, directly or indirectly, such as fear, insecurity, worry, sadness, irritability, boredom, feelings of loneliness, lack of energy, feelings of hopelessness, anxiety, sedentary lifestyle, stress, in addition to of impatience^(27,28)^.

Certainly, people were also negatively impacted on their psychological well-being, causing behavioral and emotional problems and negative feelings, especially during critical periods of the pandemic. Information about these reactions indicates the seriousness and severity of the situation experienced in the face of developments associated with this new virus.

### Study limitations

Limitations in this study were the context and moment of the COVID-19 pandemic crisis, considering that not all people were vaccinated during the period during which the research was carried out. Furthermore, social isolation restrictions and other measures to prevent the spread of the virus created some obstacles to developing the study. These aspects limited the number of participants and prolonged the study time. It was not possible to return the transcripts to participants and provide feedback on the results.

### Contributions to health

The results of this study present relevant information for the context of care for chronically ill children and adolescents, bringing important aspects that permeate their daily lives and their experience in the face of the COVID-19 pandemic.

The discussions presented about the negative feelings evidenced by participants in the face of social isolation as well as the coping and adaptation strategies used to alleviate suffering during this period, can help health professionals in the foundation of their work process, aiming at comprehensive care for children and adolescents with chronic illnesses.

This is valuable because, in addition to the weaknesses imposed by chronic illness, these professionals need to envision a context that not only involves the physical body, but also the psychological, social and spiritual dimensions. Thus, based on the perception of these experiences and strategies, the health team was able to reorganize the planning of their actions, including guidance to family members about their importance with children and adolescents in times of crisis, promotion of practices that alleviate suffering and elaboration of sensitive assistance to listen to this child audience’s needs.

## FINAL CONSIDERATIONS

Children’s and adolescents’ experiences were observed through drawings as a mark of contemporary times, highlighting, through subjective aspects, a disease that hit the world and forced everyone to stay indoors. This lived aspect was reflected among participants, and brought to the fore the abrupt disruption of routines and, above all, the impediment to playing.

It was also presumed to observe that everyday life, full of restrictions, introduced coping strategies perceived in different ways, but seen as positive and adaptive in the face of an adverse experience, in which the stressor occurred as a result of the pandemic. Thus, understanding the experiences and coping strategies of children and adolescents with chronic illness demonstrated the expression of creative imagination, incorporated by subjective components, which brings to light an approximation with the reality perceived and interpreted in a context of the COVID-19 pandemic. Finally, it is suggested that this study be replicated post-pandemic, in order to understand the longer-lasting repercussions among participants.

## Supplementary Material

0034-7167-reben-76-s2-e20230045-suppl01

0034-7167-reben-76-s2-e20230045-suppl02

0034-7167-reben-76-s2-e20230045-suppl03

0034-7167-reben-76-s2-e20230045-suppl04

## Data Availability

https://doi.org/10.48331/scielodata.8CGEIP
