# ERRATUM

**DOI:** 10.1590/0034-7167.20247701e04

**Published:** 2024-02-26

In the article “Coping strategies for chronically ill children and adolescents facing the COVID-19 pandemic”, with DOI number: https://doi.org/10.1590/0034-7167-2023-0045, published in Revista Brasileira de Enfermagem, 2023;76(Suppl 2):e20230045, in the author:

Where it read:

**Edna Maria Campelo Chaves^I^**

**ORCID: 0000-0001-9658-0377**

**How to cite this article:**

Custódio LL, Costa DCCO, Menezes CPSR, Figueiredo SV, Maia JA, Jorge MSB. Coping strategies for chronically ill children and adolescents facing the COVID-19 pandemic. Rev Bras Enferm. 2023;76(Suppl 2):e20230045. https://doi.org/10.1590/0034-7167-2023-0045

It reads:

**Edna Maria Camelo Chaves^I^**

**ORCID: 0000-0001-9658-0377**

**How to cite this article:**

Custódio LL, Costa DCCO, Menezes CPSR, Figueiredo SV, Maia JA, Jorge MSB, et al. Coping strategies for chronically ill children and adolescents facing the COVID-19 pandemic. Rev Bras Enferm. 2023;76(Suppl 2):e20230045. https://doi.org/10.1590/0034-7167-2023-0045

