# Supplementary material for: Coping strategies for chronically ill children and adolescents facing the COVID-19 pandemic
Source: Rev Bras Enferm. 2023 Dec 8;76(Suppl 2):e20230045. doi: 10.1590/0034-7167-2023-0045 (PMC10704693; doi:10.1590/0034-7167-2023-0045)
Supplement: 0034-7167-reben-76-s2-e20230045-suppl01 [file 0034-7167-reben-76-s2-e20230045-suppl01.pdf]

CHEK LIST COREQ

Critérios consolidados para relatar pesquisa qualitativa			
Nº item	Tópico	Perguntas/ descrição do guia	Resposta
Domínio 1: equipe de pesquisa e reflexividade			
	Características pessoais		
1	Entrevistador/facilitador	Qual autor (autores) conduziu a entrevista ou o grupo focal?	Os dados foram coletados por **uma pesquisadora**, em processo de doutoramento em saúde coletiva, que passou por treinamento para coleta de dados em estudos qualitativos. Foi estabelecido contato inicial com os participantes, para apresentação e ocupação da pesquisadora, seus objetivos e interesse pelo estudo. A coleta dos dados ocorreu no período de abril a agosto de 2021, sendo utilizado teste piloto com três participantes, cujos dados foram descartados.
2	Credenciais	Quais eram as credenciais do pesquisador? Exemplo: PhD, médico.	Os dados foram coletados por uma pesquisadora, em **processo de doutoramento em saúde coletiva**, que passou por treinamento para coleta de dados em estudos qualitativos. Foi estabelecido contato inicial com os participantes, para apresentação e ocupação da pesquisadora, seus objetivos e interesse pelo estudo. A coleta dos dados ocorreu no período de abril a agosto de 2021, sendo utilizado teste piloto com três participantes, cujos dados foram descartados.
3	Ocupação	Qual a ocupação desses autores na época do estudo?	Os dados foram coletados por uma pesquisadora, em processo de **doutoramento em saúde coletiva**, que passou por treinamento para coleta de dados em

			estudos qualitativos. Foi estabelecido contato inicial com os participantes, para apresentação e ocupação da pesquisadora, seus objetivos e interesse pelo estudo. A coleta dos dados ocorreu no período de abril a agosto de 2021, sendo utilizado teste piloto com três participantes, cujos dados foram descartados.
4	Gênero	O pesquisador era do sexo masculino ou feminino?	Os dados foram coletados por uma **<u>pesquisadora,</u>** em processo de doutoramento em saúde coletiva, que passou por treinamento para coleta de dados em estudos qualitativos. Foi estabelecido contato inicial com os participantes, para apresentação e ocupação da pesquisadora, seus objetivos e interesse pelo estudo. A coleta dos dados ocorreu no período de abril a agosto de 2021, sendo utilizado teste piloto com três participantes, cujos dados foram descartados.
5	Experiência e treinamento	Qual a experiência ou treinamento do pesquisador?	Os dados foram coletados por uma pesquisadora, em processo de doutoramento em saúde coletiva, que **<u>passou por treinamento para coleta de dados em estudos qualitativos</u>.** Foi estabelecido contato inicial com os participantes, para apresentação e ocupação da pesquisadora, seus objetivos e interesse pelo estudo. A coleta dos dados ocorreu no período de abril a agosto de 2021, sendo utilizado teste piloto com três participantes, cujos dados foram descartados.
	Relacionamento com os participantes		
6	Relacionamento estabelecido	Foi estabelecido um relacionamento antes do início do estudo?	Os dados foram coletados por uma pesquisadora, em processo de doutoramento em saúde coletiva, que passou por treinamento para coleta de dados em estudos qualitativos. **<u>Foi estabelecido contato inicial</u>**

			**<u>com os participantes, para apresentação e ocupação da pesquisadora, seus objetivos e interesse pelo estudo.</u>** A coleta dos dados ocorreu no período de abril a agosto de 2021, sendo utilizado teste piloto com três participantes, cujos dados foram descartados.
7	Conhecimento do participante sobre o entrevistador	O que os participantes sabiam sobre o pesquisador? Por exemplo: objetivos pessoais, razões para desenvolver a pesquisa.	Os dados foram coletados por uma pesquisadora, em processo de doutoramento em saúde coletiva, que passou por treinamento para coleta de dados em estudos qualitativos. **<u>Foi estabelecido contato inicial com os participantes, para apresentação e ocupação da pesquisadora, seus objetivos e interesse pelo estudo</u>**. A coleta dos dados ocorreu no período de abril a agosto de 2021, sendo utilizado teste piloto com três participantes, cujos dados foram descartados.
8	Características do entrevistador	Quais características foram relatadas sobre o entrevistador/facilitador? Por exemplo, preconceitos, suposições, razões e interesses no tópico da pesquisa.	Os dados foram coletados por uma pesquisadora, em processo de doutoramento em saúde coletiva, que passou por treinamento para coleta de dados em estudos qualitativos. Foi estabelecido contato inicial com os participantes, para apresentação e ocupação da pesquisadora, seus objetivos e interesse pelo estudo. A coleta dos dados ocorreu no período de abril a agosto de 2021, sendo utilizado teste piloto com três participantes, cujos dados foram descartados.
Domínio 2: Conceito do estudo			
	Estrutura teórica		
9	Orientação metodológica e teoria	Qual orientação metodológica foi declarada para sustentar o estudo? Por	**<u>Utilizou-se a análise de conteúdo para desenhos baseada nos critérios descritos por Coutinho</u>**, em

		exemplo: teoria fundamentada, análise do discurso, etnografia, fenomenologia e análise de conteúdo.	suas cinco etapas, a saber: observação sistemática dos desenhos e temas; leitura flutuante dos conteúdos das estórias; seleção dos desenhos por semelhanças gráficas e/ou aproximação dos temas; exploração do material, identificação dos núcleos de sentido, categorização e tratamento dos resultados.
	Seleção de participantes		
10	Amostragem	Como os participantes foram selecionados? Por exemplo: conveniência, consecutiva, amostragem, bola de neve.	**A seleção dos participantes para coleta de dados ocorreu até o alcance de um grau de aprofundamento das informações quanto aos objetivos traçados. A amostra se deu por conveniência, não probabilística** e presencial, ocorrida após a aceitação e aprovação do responsável legal, bem como as crianças ou adolescentes em participar da pesquisa.
11	Método de abordagem	Como os participantes foram abordados? Por exemplo: pessoalmente, por telefone, carta ou e-mail.	A seleção dos participantes para coleta de dados ocorreu até o alcance de um grau de aprofundamento das informações quanto aos objetivos traçados. A amostra se deu por conveniência, não probabilística e **presencial,** ocorrida após a aceitação e aprovação do responsável legal, bem como as crianças ou adolescentes em participar da pesquisa.
12	Tamanho da amostra	Quantos participantes foram incluídos no estudo?	**Participaram do estudo quatro crianças e dois adolescentes (acompanhados por um responsável), de ambos os sexos** e houve duas recusas (indisposição).
13	Não participação	Quantas pessoas se recusaram a participar ou desistiram? Por quais motivos?	Participaram do estudo quatro crianças e dois adolescentes (acompanhados por um responsável), de

			ambos os sexos e **houve duas recusas por indisposição).**
	Cenário		
14	Cenário da coleta de dados	Onde os dados foram coletados? Por exemplo: na casa, na clínica, no local de trabalho.	O estudo realizado em um **ambulatório especializado no atendimento de crianças e adolescentes com doenças crônicas, em um Hospital público pediátrico, localizado em Fortaleza- CE (Brasil)**.
15	Presença de não participantes	Havia mais alguém presente além dos participantes e pesquisadores?	Participaram do estudo quatro crianças e dois adolescentes (**acompanhados por um responsável**), de ambos os sexos e houve duas recusas (indisposição).
16	Descrição da amostra	Quais são as características importantes da amostra? Por exemplo: dados demográficos, data da coleta.	Participaram da amostra **quatro crianças e dois adolescentes.** Desses, 75% das crianças e 50% dos adolescentes são do sexo feminino. A **média de idade** entre as crianças e adolescentes foi respectivamente **8,25 e 13 anos**. **Os participantes do estudo são oriundos 66,7% de outros municípios cearense e apenas 33,3% de Fortaleza, Ceará.**
	Coleta de dados		
17	Guia da entrevista	Os autores forneceram perguntas, instruções, guias? Elas foram testadas por teste-piloto?	Os dados foram coletados por uma pesquisadora, em processo de doutoramento em saúde coletiva, que passou por treinamento para coleta de dados em estudos qualitativos. Foi estabelecido contato inicial com os participantes, para apresentação e ocupação da pesquisadora, seus objetivos e interesse pelo estudo. A coleta dos dados ocorreu no período de abril a

			agosto de 2021, **<u>sendo utilizado teste piloto com três participantes, cujos dados foram descartados.</u>**<br>**<u>(..)</u>**<br><br>Foi solicitado aos participantes a confecção de um desenho, a partir **<u>da seguinte instrução:</u>** "**<u>Desenhe sobre como é para você conviver com uma doença crônica em tempos de pandemia do novo coronavírus".</u>** Após a finalização da produção, eles foram convidados a contar sobre o que haviam desenhado, seja em forma de estória ou de explicação, de modo a atribuir o seu sentido ou significado de maneira verbal. Essa aplicação aconteceu em um momento único, de maneira livre, individual, com duração média de 30 minutos.
18	Repetição de entrevistas	Foram realizadas entrevistas repetidas? Se sim, quantas?	Foi solicitado aos participantes a confecção de um desenho, a partir da seguinte instrução: "Desenhe sobre como é para você conviver com uma doença crônica em tempos de pandemia do novo coronavírus". Após a finalização da produção, eles foram convidados a contar sobre o que haviam desenhado, seja em forma de estória ou de explicação, de modo a atribuir o seu sentido ou significado de maneira verbal. **<u>Essa aplicação aconteceu em um momento único,</u>** de maneira livre, individual, com duração média de 30 minutos.
19	Gravação audiovisual	A pesquisa usou gravação de áudio ou visual para coletar os dados?	**<u>Os dados (produções escritas ou verbalizadas) foram transcritos para o Word, observando a veracidade dos depoimentos obtidos, durante os registros no diário de campo, uma vez que as gravações não foram autorizadas</u>**. Além de levar-se

			em conta o desenho como um instrumento de comunicação, com foco na verificação de elementos simbólicos que fossem significativos durante a coleta de dados.
20	Notas de campo	As notas de campo foram feitas durante e/ou após a entrevista ou o grupo focal?	Os dados (produções escritas ou verbalizadas) foram transcritos para o Word, o**<u>bservando a veracidade dos depoimentos obtidos, durante os registros no diário de campo,</u>** uma vez que as gravações não foram autorizadas. Além de levar-se em conta o desenho como um instrumento de comunicação, com foco na verificação de elementos simbólicos que fossem significativos durante a coleta de dados.
21	Duração	Qual a duração das entrevistas ou do grupo focal?	Foi solicitado aos participantes a confecção de um desenho, a partir da seguinte instrução: "Desenhe sobre como é para você conviver com uma doença crônica em tempos de pandemia do novo coronavírus". Após a finalização da produção, eles foram convidados a contar sobre o que haviam desenhado, seja em forma de estória ou de explicação, de modo a atribuir o seu sentido ou significado de maneira verbal. Essa aplicação aconteceu em um momento único, de maneira livre, individual, **<u>com duração média de 30 minutos.</u>**
22	Saturação de dados	A saturação de dados foi discutida?	**<u>A seleção dos participantes para coleta de dados ocorreu até o alcance de um grau de aprofundamento das informações quanto aos objetivos traçados</u>**. A amostra se deu por conveniência, não probabilística e presencial, ocorrida

			após a aceitação e aprovação do responsável legal, bem como as crianças ou adolescentes em participar da pesquisa.
23	Devolução de transcrições	As transcrições foram devolvidas aos participantes para comentários e/ou correção?	Evidenciou-se como limitação neste estudo, o contexto e o momento de crise pandêmica da Covid-19, tendo em vista que nem todas as pessoas estavam vacinadas no período de realização da pesquisa. Além disso, as restrições de isolamento social e outras medidas para prevenção da propagação do vírus geraram alguns entraves para o desenvolvimento do estudo. Aspectos estes que limitaram o quantitativo de participantes e prolongaram o tempo de realização do estudo. **Assim como, não foi possível a devolução das transcrições aos participantes** e um momento de feedback sobre os resultados. No entanto, todos os dados foram entregues ao CEP do Hospital.
Domínio 3: Análise e resultados			
24	Número de codificadores de dados	Quantos foram os codificadores de dados?	Quanto à análise do instrumento Desenhos-estória de Trinca[12], importante no processo de observação clínica, percebeu-se que a proposta resultou em movimentos positivos pelos participantes no desenvolvimento e expressão de sua atividade. Dessa forma, foi a partir das produções derivadas da coleta de dados que **emergiram duas categorias,** apresentadas logo em seguida.
25	Descrição da árvore de codificação	Os autores forneceram uma descrição da árvore de codificação?	**1.Situações vivenciadas por crianças e adolescentes em tempo da Covid-19**<br>**2. Estratégias de enfrentamento nas situações de crise nas crianças e adolescentes em seu processo**

			**de adoecimento crônico durante a pandemia da Covid-19**
26	Derivação de temas	Os temas foram identificados antecipadamente ou derivados dos dados?	Quanto à análise do instrumento Desenhos-estória de Trinca[12], importante no processo de observação clínica, percebeu-se que a proposta resultou em movimentos positivos pelos participantes no desenvolvimento e expressão de sua atividade. Dessa forma, **foi a partir das produções derivadas da coleta de dados que emergiram duas categorias**, apresentadas logo em seguida.
27	Software	Qual software, se aplicável, foi usado para gerenciar os dados?	**Não aplicável.**<br>Os dados (produções escritas ou verbalizadas) foram transcritos para o Word, observando a veracidade dos depoimentos obtidos, durante os registros no diário de campo, uma vez que as gravações não foram autorizadas.
28	Verificação do participante	Os participantes forneceram feedback sobre os resultados?	Evidenciou-se como limitação neste estudo, o contexto e o momento de crise pandêmica da Covid-19, tendo em vista que nem todas as pessoas estavam vacinadas no período de realização da pesquisa. Além disso, as restrições de isolamento social e outras medidas para prevenção da propagação do vírus geraram alguns entraves para o desenvolvimento do estudo. Aspectos estes que limitaram o quantitativo de participantes e prolongaram o tempo de realização do estudo. Assim como, **não foi possível** a devolução das transcrições aos participantes **e um momento de feedback sobre**

			**<u>os resultados.</u>** No entanto, todos os dados foram entregues ao CEP do Hospital.
	Relatório		
29	Citações apresentadas	As citações dos participantes foram apresentadas para ilustrar os temas/achados? Cada citação foi identificada? Por exemplo, pelo número do participante.	O estudo atendeu a Resolução nº 466 de 12 de dezembro de 2012[11] que trata das questões éticas envolvendo seres humanos. Todos os participantes assinaram os termos de Consentimento e Assentimento Livre e Esclarecido e tiveram seu anonimato preservado. **<u>Visto que, foram identificados com a letra C, para criança e A para adolescente, seguido de uma ordem numérica, idade em anos e letra F para feminino e M para masculino (ex: C1, 9a, M; A1,12a, F).</u>**
30	Dados e resultados consistentes	Houve consistência entre os dados apresentados e os resultados?	**<u>A consistência da pesquisa se deu por meio do instrumento Desenhos-estória de Trinca[12], importante no processo de observação clínica, percebeu-se que a proposta resultou em movimentos positivos pelos participantes no desenvolvimento e expressão de sua atividade.</u>** Dessa forma, foi a partir das produções derivadas da coleta de dados que emergiram duas categorias, apresentadas logo em seguida.
31	Clareza dos principais temas	Os principais temas foram claramente apresentados nos resultados?	**<u>1. Situações vivenciadas por crianças e adolescentes em tempo da Covid-19</u>** A leitura dos desenhos possibilitou a pesquisadora compreensão psicológica da realidade dos participantes, que esboçaram suas angústias, medos e percepções da realidade no momento pandêmico,

			elencada na primeira categoria. O quadro 1 apresenta o relato dos sentimentos de alguns participantes e desenhos em relação às situações vivenciadas pelos participantes durante a pandemia Covid-19.<br>**2. Estratégias de enfrentamento nas situações de crise nas crianças e adolescentes em seu processo de adoecimento crônico durante a pandemia da Covid-19.**<br>Sobre as estratégias de enfrentamento, foi possível perceber que as táticas utilizadas foram diferentes por cada participante. Enquanto um expressou que recorreu à espiritualidade, com a exposição de um anjo (foco na espiritualidade, emoção), outra trouxe o uso de suas habilidades para enfrentar situações durante a covid-19 (foco no problema) e outra ainda, apresentou a sua família (foco na emoção), como forma de recorrência, conforme se encontra exposto nos desenhos.<br>O quadro 2 apresenta a materialização dos sentimentos de alguns participantes, por meio do desenho em relação às estratégias de enfrentamento nas situações de crise nas crianças e adolescentes em seu processo de adoecimento crônico durante a pandemia da Covid-19.
32	Clareza de temas secundários	Há descrição dos diversos casos ou discussão dos temas secundários?	**Não se aplica.**
