# Supplementary material for: Coping strategies for chronically ill children and adolescents facing the COVID-19 pandemic
Source: Rev Bras Enferm. 2023 Dec 8;76(Suppl 2):e20230045. doi: 10.1590/0034-7167-2023-0045 (PMC10704693; doi:10.1590/0034-7167-2023-0045)
Supplement: 0034-7167-reben-76-s2-e20230045-suppl02 [file 0034-7167-reben-76-s2-e20230045-suppl02.pdf]

FORMULÁRIO PARA CRIANÇAS/ADOLESCENTES

I - Dados de identificação

Idade: 11 anos

Sexo: ( ) Feminino (x) Masculino ( ) ______________

Escolaridade: ( ) analfabeto (x) Fundamental ________ ( ) Médio __________

II - Instrução para o desenho:

Para confecção de um desenho, foi repassado a seguinte informação:

"Desenhe sobre como é para você conviver com uma doença crônica em tempos de pandemia do novo coronavírus".

Após a finalização da produção, as crianças ou adolescentes foram convidados a contar sobre o que haviam desenhado, seja em forma de estória ou de explicação, a expressão era livre, de modo a atribuir o seu sentido ou significado de maneira verbal

A criança que produziu a figura 2 (Não posso sair!), fez o desenho dele desenhou dentro de casa, no local onde seria a porta, ele colocou um X, e ao lado da casa, uma bicicleta. Ao ser solicitado para explicar sobre seu desenho, ele se colocou em uma postura séria e mais fechada, parecendo indignado pelo que estava vivendo:

Esse sou eu, estou super triste oh, porque estou dentro de casa, sem poder sair, sem poder fazer o que mais gosto de minha vida que é brincar. Vivo preso em casa. Não posso jogar, nem andar de bicicleta. Respirou fundo e continuou: 'Fique em casa'. É muito é chato isso. Neh, pude nem andar. Ficava doido pra ir atrás dos meus amigos, mas minha mãe nem deixava. Antes, todo dia andava de bicicleta, brincava com meus amigos, agora não posso. Tomara que 'isso' (doença) vá logo embora. Pior coisa da minha vida .
