# Supplementary material for: Coping strategies for chronically ill children and adolescents facing the COVID-19 pandemic
Source: Rev Bras Enferm. 2023 Dec 8;76(Suppl 2):e20230045. doi: 10.1590/0034-7167-2023-0045 (PMC10704693; doi:10.1590/0034-7167-2023-0045)
Supplement: 0034-7167-reben-76-s2-e20230045-suppl03 [file 0034-7167-reben-76-s2-e20230045-suppl03.pdf]

FORMULÁRIO PARA OS PAIS/RESPONSÁVEIS

I - **Dados de identificação**
D.N.: ___/___/___ Idade: _________
( ) pai ( ) mãe ( ) responsável: _________________
Endereço:_________________________________ cidade: ______________
Telefone para contato:_____________________
D.N da criança/adolescente.: ___/___/___ Idade da criança/adolescente: _____

II - Bloco A (sócio demográficos)
1. Sexo: ( ) Feminino ( ) Masculino ( ) ______________
2. Escolaridade: ( ) analfabeto ( ) Fundamental ________ ( ) Médio __________
( ) Superior_________________
3. Estado civil: ( ) Solteiro ( ) Casado ( ) Viúvo ( ) Separado ( ) outros:______
4. Cor referida: ( ) branca ( ) parda ( ) preto ( ) amarelo ( ) indígena ( ) ___________
5. Procedência: ( ) capital ( ) interior ( ) outro ________________
6. Religião: ( ) católica ( ) Protestantes/evangélica ( ) espírita ( ) judaísmo
( ) Hinduísmo ( ) macumba ( ) matriz africana ________ Islamismo ( ) ________

- Renda, atividade profissional, estrutura familiar e moradia
7. Trabalha: ( ) Sim ( ) Não ( ) aposentado
Ocupação: _____________________
8. Renda familiar: ____________________ salários(s)
9. Recebe algum benefício: ( ) não ( ) sim ___________________
10. Quantidades de membros na residência:_______________ pessoa(s)
11. Com quem mora: ( ) Pais ( ) Avós ( ) Companheiro ( ) Filhos ( ) Outros:___________
12. Quantos compartimentos na casa: ______ quais: _________________________
13 Existência de saneamento básico: ( ) sim ( ) não

III - Bloco B (epidemiológicos)

– Dados clínicos da doença
14. Qual doença seu filho (a) tem?
( ) Diabetes Mellitus ( ) Doença Hepatica ( ) doença cardíaca ( ) câncer
( ) outras qual?____________________________________
15. Apresenta complicações: ( ) Sim ( ) Não
Especifique: ____
____
16. Faz tratamento: ( ) sim ( ) não Local: __________________________________
17. O que você sabe sobre a doença do seu filho (a): ( ) conhece ( ) não conhece. Explique:
____
____

- Antecedentes da doença e familiares

18. Qual a idade que a criança/adolescente tinha quando realizou o diagnóstico? ____
19. Qual a situação em que ocorreu o diagnóstico? ____

20. Qual o exame realizado? ____

21. Familiares com a mesma DCNT (prevalência): ( ) Sim ( ) Não. Quem:_____________

IV – Dados relacionados adoecimento durante a pandemia
22. Presença de manifestações clínicas: ( ) não ( ) sim
Quais:____
23. Tipos e durações: ( ) nunca ( ) as vezes ( ) muitas vezes _____________
24. Presença de outra morbidade: __________________________________

IV – Bloco C (Estratégias durante a pandemia de Covid-19)
25. O seu filho (a) necessitou de cuidados especiais por causa de sua condição de Saúde durante a pandemia? ( ) Sim ( ) Não. Se sim, quais? ____

26. Procurou algum serviço de saúde durante a pandemia? ( ) não ( ) sim
Se sim, Qual a unidade procurada: ( ) atenção primaria/ posto ( ) UPA ( ) hospital _________________( ) outros: ____
Quantas vezes você procurou pelos serviços de saúde? __________________________
26. Como ficaram sabendo da Unidade a recorrer?
____

27. Qual meio de transporte foi utilizado para chegar a Unidade de Saúde
( ) carro próprio ( ) a pé ( ) ônibus ( ) úber/taxi ( ) carona _____________
28. Quem geralmente acompanha a criança ou o adolescente? ____

29. Fizeram marcação de consulta? ( ) não ( ) sim
30. O que os profissionais fizeram com seu filho (a): ( ) medicação ( ) internação ( ) outro tipo de cuidado ____

____

31. Como ocorreu para a obtenção de medicamentos?
____
____
____

32. O Senhor (a) ficou satisfeito com o atendimento? ( )não ( ) sim
32. Em sua opinião, quais as facilidades e dificuldades encontradas para se ter acesso ao serviço de saúde?
____
____
____
____
____
