# Supplementary material for: Coping strategies for chronically ill children and adolescents facing the COVID-19 pandemic
Source: Rev Bras Enferm. 2023 Dec 8;76(Suppl 2):e20230045. doi: 10.1590/0034-7167-2023-0045 (PMC10704693; doi:10.1590/0034-7167-2023-0045)
Supplement: 0034-7167-reben-76-s2-e20230045-suppl04 [file 0034-7167-reben-76-s2-e20230045-suppl04.pdf]

Produções de crianças e adolescentes participantes da pesquisa

1) **Situações vivenciadas por crianças e adolescentes em tempo de Covid-19**

Nessa categoria tiveram representações de realidades vividas dentro de casa, conforme mostram os desenhos de três participantes.

O primeiro foi um adolescente que se desenhou dentro de sua casa, tendo em uma de suas mãos um papel e na outra mão um lápis. Portas e janelas estão fechadas, e ao lado da casa aparece uma árvore e do outro tem um poste.

Figura 1 - No tempo da pandemia

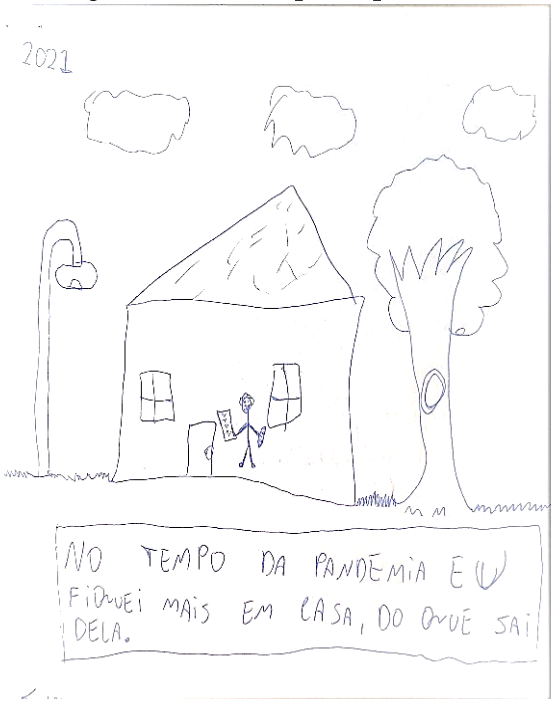

Fonte: A1, 13 anos.

Ao ser convidado a explicar, o adolescente, A1, de 13 anos de idade, identificou todos os elementos presentes em seu desenho. Depois falou: "Dentro dessa casa sou eu, estou dentro dela, porque não pude sair. Não só eu, mas todo mundo [...] tive até que estudar em casa. Não pude nem brincar". Escrevi nessa frase abaixo: No tempo da pandemia Eu fique mais em casa do que sai.

O segundo participante, foi uma criança, C1, contava com 11 anos. Também se desenhou dentro de casa, no local onde seria a porta, ele colocou um X, e ao lado da casa, uma bicicleta.

Figura 2 - Não posso sair!

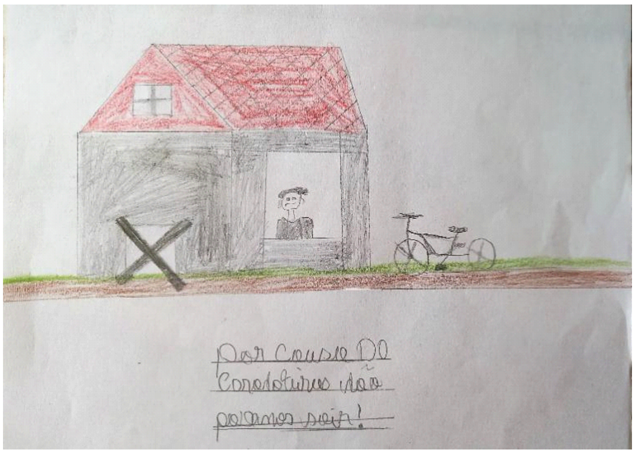

Fonte: C1, 11 anos.

Para além do desenho, C1 conseguiu se expressar. Colocou-se em uma postura séria e mais fechada, parecendo indignado pelo que estava vivendo:

> Esse sou eu, estou super triste oh, porque estou dentro de casa, sem poder sair, sem poder fazer o que mais gosto de minha vida que é brincar. Vivo preso em casa. Não posso jogar, nem andar de bicicleta [...] ‘Fique em casa’. É muito é chato isso. Neh, pude nem andar. Ficava doido pra ir atrás dos meus amigos, mas minha mãe nem deixava. Antes, todo dia andava de bicicleta, brincava com meus amigos, agora não posso. Tomara que ‘isso’ (doença) vá logo embora. Pior coisa da minha vida (C1).

A seguir, o terceiro participante realizou um desenho mais animado e colorido, parecendo uma espécie de duas representações. Conforme aparece na figura abaixo.

Figura 3 - Em casa

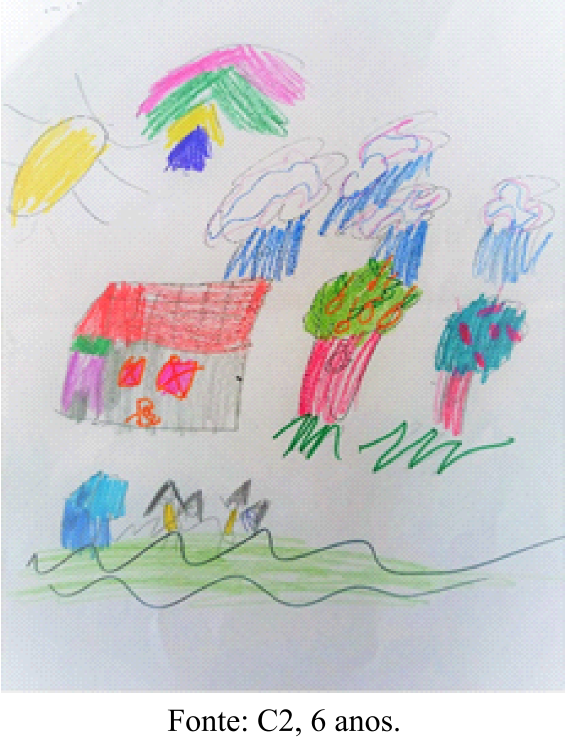

Fonte: C2, 6 anos.

Quando solicitada a explicar a sua produção, C2 apontou inicialmente para casa maior, na parte acima da folha, e realizou a seguinte explicação:

> "ai é onde eu moro. Minha casa. Mas agora tô dentro de casa e não posso brincar. Você vê que tá fechado? Pois é, minha mãe fecha pra ninguém sair [...] Fico um tempão só em casa. Brincar que é bom, nem nada. Só vivo lá (casa)" (C2).

Com o olhar e a voz um pouco entristecida, apontou para a parte inferior da folha, mostrando o desenho em torno da casa menor, toda azul, continuou sua fala: "Aqui antes era eu, era assim, brincava que só com minhas amigas. Agora não pode. Gostava um montão assim" [...]. Nesse momento, a criança abriu os braços bem abertos. Acredita-se que houve uma tentativa de mostrar ou representar "um montão assim", como uma grande quantidade. Para finalizar, a criança complementou ainda: "fiz esses riscos de preto nessa parte, porque não posso mais brincar com elas (outras crianças), ai tenho que ficar em casa".

2) **Estratégias de enfrentamento nas situações de crise nas crianças e adolescentes em seu processo de adoecimento crônico durante a pandemia de Covid-19.**

Nessa categoria, uma criança faz o seu desenho tendo a exposição de um anjo, conforme se encontra exposto nos desenhos.

Figura 4 - O anjo de Jesus

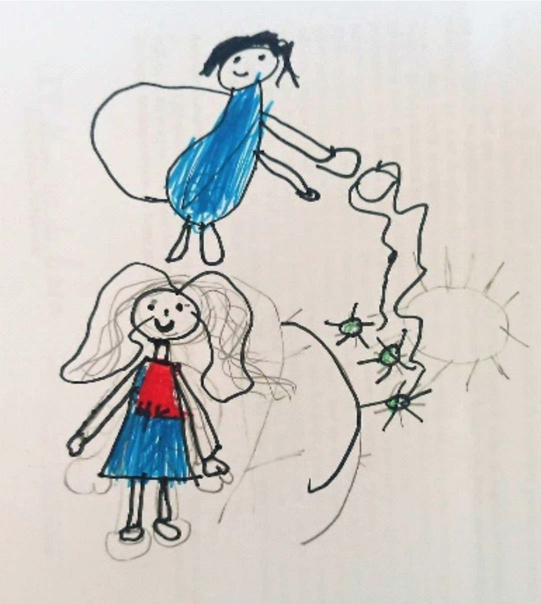

Fonte: C_3, 9 anos.

A criança C3 de maneira breve e bem objetiva olhou para o seu desenho e foi logo explicando a sua obra: "aqui sou eu e o anjo de Jesus, em cima de mim. Ele tá matando o coronavírus. Ele tá dando proteção pra mim, pra eu não pegar o vírus. Eu peço a Jesus pra mim proteger. Faço sempre isso e tá dando certo".

A figura 5, foi a de uma adolescente que outra trouxe o uso de suas habilidades para enfrentar situações durante a covid-19.

Figura 5 - Despertando desejos.

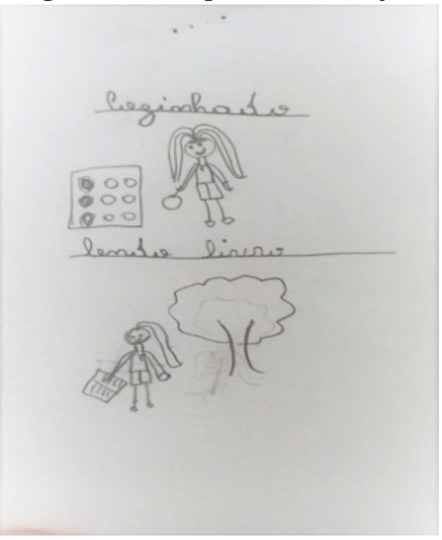

Fonte: A2, 13 anos.

Sobre seu desenho, A2 informou: “Essa sou eu”, apontando para o desenho superior disse: “aqui estou na cozinha”. Depois apontou para o de baixo e continuou: “aqui eu lendo perto de uma árvore no meu quintal”. Ao ser solicitada para explicar melhor, A falou:

> Na pandemia fiquei mais em casa, ai tive o desejo de ler e aprender a cozinhar. Não tinha nada pra fazer [...] Aprendi a fazer cuscuz de frigideira, omelete, biscoito com aveia e outras comidas mais. Também eu adoeci e não podia comer qualquer coisa. Eu pesquisava na internet e fazia. Pra mim foi legal. Outra coisa boa foi que aprendi a ler, pois não gostava, mas não era livro da escola não. Era livro com outras leituras. Sabia que antes eu não gostava. Até minha mãe se empolgou (risos), ela comprou dois livros pra mim e eu li. Foi o que mais fiz na pandemia (A2).

Por último, na figura abaixo, viu-se a representação de uma família de mãos dadas, dentro de um possível coração.

Figura 6 - Amor que protege

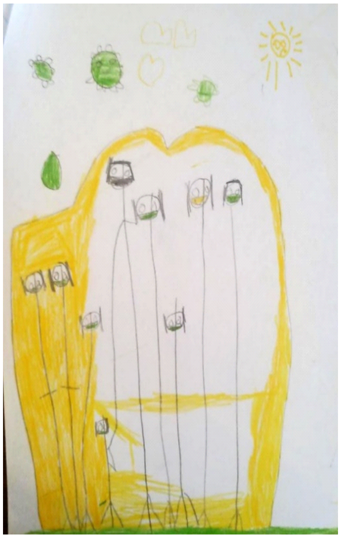

Fonte: C_4, 7 anos.

C4 esclareceu sua arte, apontando para parte amarela com pessoas dentro e comunicou:

> Desenhei toda minha família. Aqui tem a minha tia, padrinho e os primos, mas tem também meu pai, minha mãe e meus irmãos. Estamos cobertos no coração que protege. Família tá toda de máscara pra se proteger. Coração para proteger, ele é de pedra, é duro. Ninguém consegue abrir. É duro que nem uma rapadura [...] O covid, de verde, tá fora do coração [...] Esse é o sol (amarelo) ele tá triste porque queria caminhar e destruir os coronavírus, mas ele não conseguiu (C4).

Adverte-se que a criança não conseguiu finalizar a pintura, porque chegou o momento da consulta e a mesma foi levada pela mãe sem poder finalizar a sua obra.
